# Supplementary figures and images for: HuR (ELAVL1) regulates the CCHFV minigenome and HAZV replication by associating with viral genomic RNA
Source: PLoS Negl Trop Dis. 2024 Sep 30;18(9):e0012553. doi: 10.1371/journal.pntd.0012553 (PMC11466401; doi:10.1371/journal.pntd.0012553)

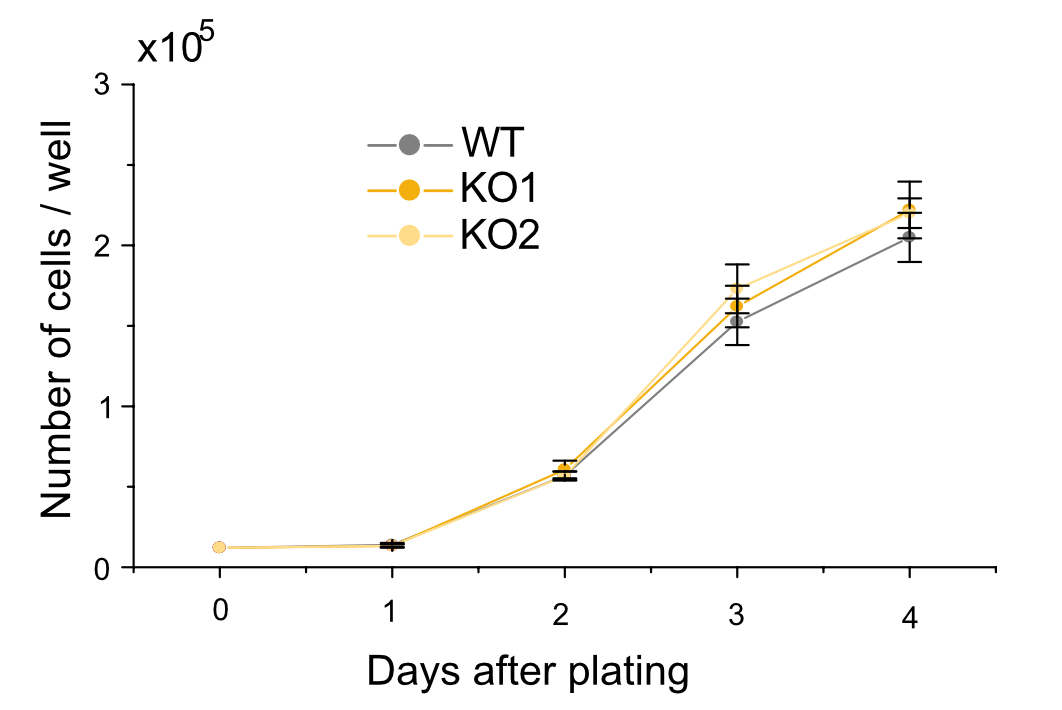

Supplement: S1 Fig — 1.2 × 10^5^ of WT, HuR KO1 and KO2 RAW264.7 cells were plated on 24 well plate and the number of cells per well was counted on the indicated day. (TIFF) [file pntd.0012553.s004.tiff]

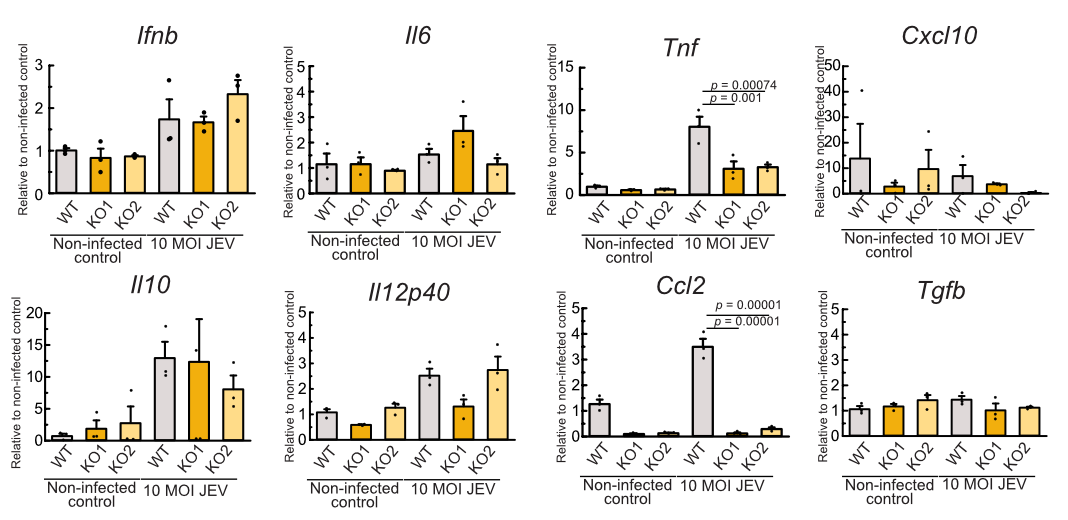

Supplement: S2 Fig — WT and HuR KO RAW264.7 cells were infected with 10 MOI JEV, and cytokine gene expression was measured at 9 h after infection by real-time PCR. Gene expression was calculated as fold increase compared to uninfected control WT cells. (TIFF) [file pntd.0012553.s005.tiff]

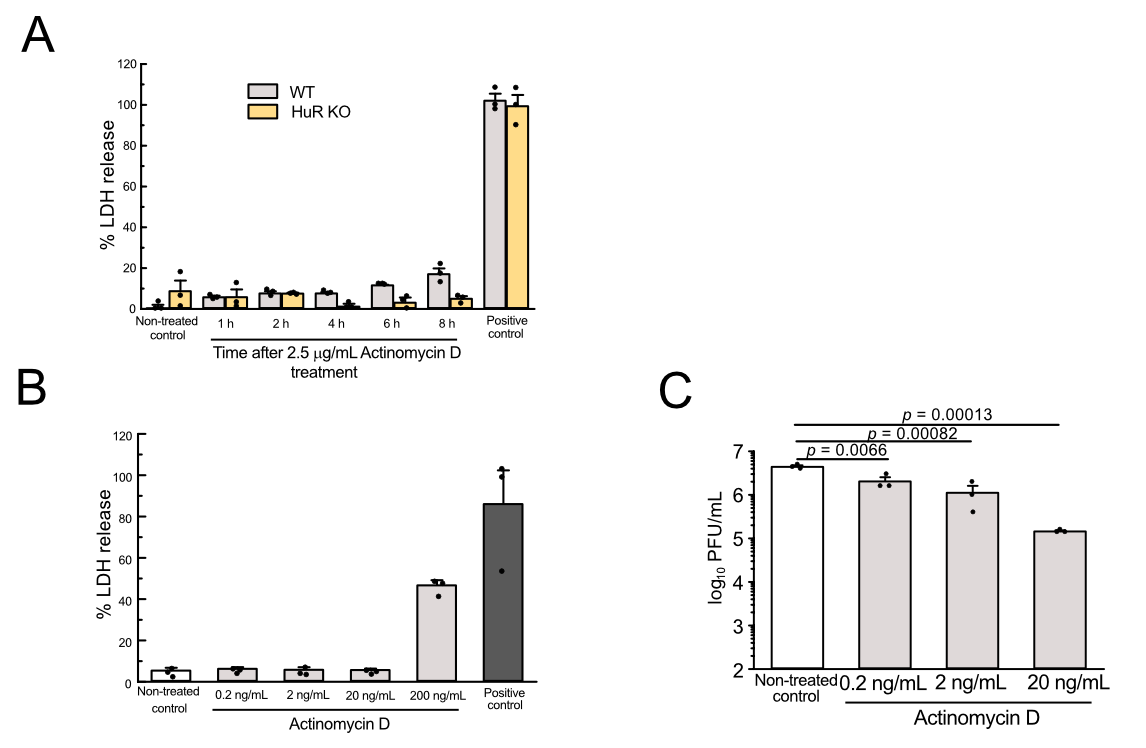

Supplement: S3 Fig — A, WT and HuR KO RAW264.7 cells were treated with 2.5 μg/mL of actinomycin D and LDH release at the indicated time points was measured. B, RAW264.7 cells were treated with the indicated concentration of actinomycin D for 48 h and LDH release in the supernatant was measured. C, RAW264.7 cells were infected with 0.1 MOI HAZV in the presence of the indicated concentration of actinomycin D. The virus titer was measured at 48 h post infection. (TIFF) [file pntd.0012553.s006.tiff]

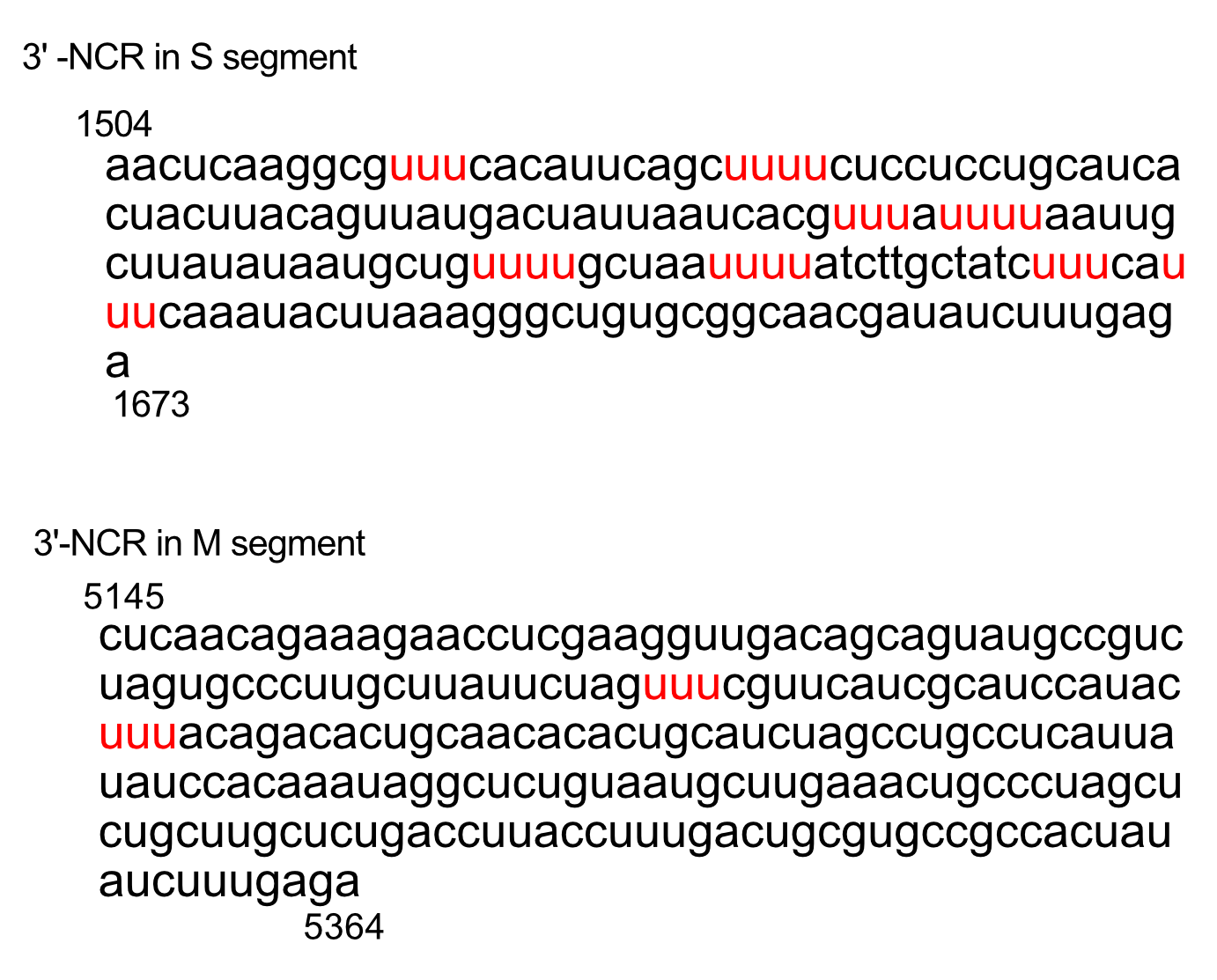

Supplement: S4 Fig — The AU-rich sequences that are not located in the panhandle structures were highlighted in S and M segments of 3’-NCR. (TIFF) [file pntd.0012553.s007.tiff]

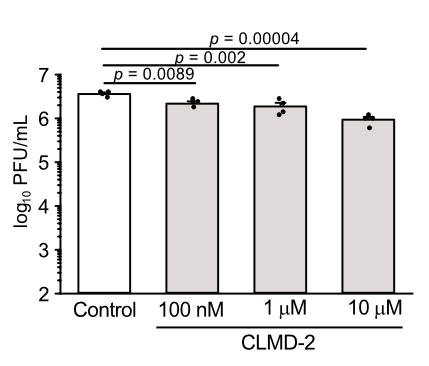

Supplement: S5 Fig — RAW264.7 cells were treated with CLMD-2 after infection with 0.1 MOI HAZV. The virus titer in the supernatant at 2 days post infection was measured by the plaque assay. (TIFF) [file pntd.0012553.s008.tiff]
