# Supplementary material for: HuR (ELAVL1) regulates the CCHFV minigenome and HAZV replication by associating with viral genomic RNA
Source: PLoS Negl Trop Dis. 2024 Sep 30;18(9):e0012553. doi: 10.1371/journal.pntd.0012553 (PMC11466401; doi:10.1371/journal.pntd.0012553)
Supplement: Attachment — Submitted filename: Response to reviewers_240827.docx [file pntd.0012553.s009.docx]

We thank the reviewer for his/her helpful comments which have allowed us to improve the paper. The following changes have been made.

Reviewer #1:

**Methods**
Reviewer #1: Objectives of the study are not clearly articulated and the aim of the work is not well indicated in the introduction. The picture of the study became clear only after the reading of the discussion. Authors used several cellular and virus models but a better connection between the experiments should be reported. In addition, three different viruses were compared in the first part of the work showing a different behavior in their replication HuR-mutated cells. Thus, to support the specific mechanism of action of HuR on HAZV replication, in some experiments all the three viruses should be included, as reported in the general comments. Some technical details are lacking, as indicated in the general comments. The statistical analyses applied to the results is sufficient.

**Results**

Reviewer #1: The analyses plan was poorly described, thus the match of the presented analyses with the analyses plan cannot be evaluated. Not all the results are clearly presented and some controls are lacking as well as additional analyses to support authors’ conclusions increasing the strength of the study. Something is lacking to have a complete picture of the phenotype observed in the mutated cell lines linking the modulation of the immune response to HAZV replication.

The quality of images is sufficient for clarity.

**Conclusion**

Reviewer #1: Conclusion are partially supported by the results and not limitations are described.

The advance of the knowledge and the potential applications are reported, as well as the potential application for the development of therapeutics.

→We apologize for not clearly describing the introduction, methods, or experimental design. Based on the reviewer's comments, we have addressed the point-by-point comments in the following sections.

**Editorial and Data Presentation Modifications?**

Reviewer #1: Minor comments:
In the section “Introduction” avoid the repetition of the segmented nature of the genome. Write better the fact that CCHFV and HAZV belong to the same genus and share many structural and biological characteristics.

→Line 83-84, we have deleted the sentence for the segmented nature of the genome and added “Hazara virus (HAZV) and CCHFV belong to the same genus, and share many structural and biological characteristics.”

Line 76: the M segment encodes the precursor that originates the mature glycoproteins of the viral envelope (Gn and Gc) and some additional proteins.

→Line 92-94, we edited the sentence as “M segment encodes the precursor that originates the mature glycoproteins of the viral envelope (Gn and Gc) and some additional proteins”

Lines 81-83: it is not clearly exposed the immune-regulation and its link to the next section

→Lines 98-99, we mentioned about cytokine production by CCHFV infection to connect immune-regulation with references.

Line 102: clarify the mean of “sequence method”

→Line 122, “sequence method” have been replaced to “RNA sequences”.

Line 126: the reference 21 is not appropriate for this citation

→Line 168 in the revised manuscript, we have deleted reference 12 in Materials and methods.

Line 133: the word “mounted” is not appropriate

→Line 176, we have replaced “mounted” to “added”.

Lines 146-158: change “sense” with “forward”

→As #2 reviewer suggested, the primer information has been moved to S2 table. We have changed “sense” with “forward” in S2 table.

Line 178, the word “harvested” is not appropriate

→Line 228, we have replaced “harvested” with “plated”.

Lines 235-236: Sentence is wrong. Cells were infected with the viruses. Please rewrite.

→We have deleted this sentence from the revised manuscript.

**Summary and General Comments**

Reviewer #1: The manuscript by Ikegawa et al reports the identification of HuR as a host protein required for CCHFV and HAZV replication in cells by its ability to bind and stabilize viral RNA.

The topic is of interest providing a new piece of knowledge on the virus-host interaction. However, there are important issues that need to be addressed.

The abstract and the summary contain non-homogeneous information and should be rewrite. In addition, the abstract start with HAZV and its role as model for CCHFV. In the introduction no info on HAZV were reported. I suggest to add a paragraph on HAZV. In addition, more details on the CCHF pathogenesis should be introduce to better understand the correlation between the different parts of the introduction (immune activation, target cells of CCHFV, etc).

In the section “material and methods” some details are missing, such as: the indication of JEV and TBEV strains, and their production; details on the PCRs protocol; the antibodies used in the western blotting; the protocol of poly(I:C) stimulation.

We understand the concerns raised by the reviewers. Each of these points is described below.

→We have rewritten the abstract and summary, and the editing has been highlighted in red letter in the revised manuscript.

→Line 76-79, we have added CCHF pathogenies.

→Line 85-90, we have included research of HAZV related to revealing molecular pathogenesis and immune activation. We also have included additional reference (doi: 10.1128/jvi.01698-23)

→Lines 174-176, we have added the strain information of JEV and TBEV with references.

→Lines 187-198, we have added the protocol of RT-PCR.

→Lines 145-147, we have added the protocol of poly(I:C) stimulation.

→Lines 215-225, we have added the antibodies used in the western blotting and dilution ratio of these antibodies. We have generated an antibody against HAZV N for revised experiment in Fig 4E and G and have included the brief method for anti-N antibody.

**Regarding the “results” section:**

Since viruses are strongly affected by cell metabolism and growth, data on the proliferation of mutated and wild type cells should be reported.

→1.2 x 10^5^ of wild type, HuR KO1 and KO2 RAW264.7 cells were plated on 24 well plate and the number of cells per well was counted in Supplemental Figure 1. Number of cells in both wild type and KO cells was not significantly altered. We have described about the proliferation in line 285-287.

In Tab S1 there are three genes that, after inactivation, are associated to a higher level of inhibition of HAZV in comparison to HuR. Why was HuR selected instead of the other three genes?

→We have shown that HuR regulates the innate immune response by binding to the 3'-NCR of host target mRNA, and we are interested in the regulatory role of HuR. To investigate whether HuR regulates the stability of exogenous RNA in addition to the host mRNA, we selected HuR for this study. We have included this in lines 280-284. As the reviewer indicated, other hits potentially regulate HAZV and CCHFV RNA stability or replication. In the future, we will investigate the role of molecular mechanisms in other RNA-binding proteins.

Lines 242-245. Regarding the mechanisms of HAZV immune-evasion, the N protein is also involved (10.3390/v14091965). This information should be included and dis^1^cussed.

→Thank you for this suggestion. Reviewer #1 instructed us to add the reference in lines 242-245 in the old manuscript, and Reviewer #2 also commented on the same lines 242-245 to mention the immune response. We have changed the sentence as “CCHFV and HAZV infection caused activation innate immune response to induce expression of cytokine genes.” in lines 299-301 in the revised manuscript, based on Reviewer #2's comment. The sentence related to HAZV/CCHFV OTU domains was omitted from the Result section; instead, we have discussed HAZV/CCHFV OTU domains and N protein with the reference (10.3390/v14091965) in the discussion at lines 463-469.

Figure 1D. Since TBEV and JEV are differentially affected by the inactivation of HuR, to better support the specific down modulation of HAZV replication, the kinetics of TBEV and JEV should be included as comparison.

→We measured the kinetics of virus titer of TBEV and JEV for 5 days and included in Figure 1D. The TBEV titer between WT and HuR KO cells was not significantly altered, except at 72 h post infection. JEV titer in HuR KO cells was higher than that in WT cells at 48 and 72 h post infection. It is unclear why the TEBV titer at 72 h and JEV virus titer at 48 and 72 h were increased in HuR KO cells, HuR deficiency differentially affected in viral replication. We have added these points in line 287-295.

Results in figure 2A lack of a negative control represented by the inactivated virus to evaluate the effect of the productive infection (with virus replication) in comparison to the input of an inactive virus.

→We understand the concerns raised by the reviewers. HAZV was exposed to 30 W UV light (CRF/UV-30A) for 30 min for inactivation, and cytokine gene expression at 9 h in RAW264.7 cells after stimulation with inactivated HAZV was included in Figure 2A. RAW264.7 cells treated with the same number of inactivated HAZV with 1 MOI HAZV as negative control. Increase of *Ifnb*, *Il6*, *Tnf* and *Cxcl10* was not detected after the addition of UV treated HAZV. In addition, copy number of HAZV S segment inside cell was calculated in Figure 2B. We have added these points in lines 305-311. UV treatment has been mentioned in the materials and methods in line 180-181.

Lines 257-258. The sentence stands that “These results indicated that the innate immune response against HAZV infection was reduced by HuR-deficiency.” In this scenario, the deletion of HuR should increase the replication of HAZV. The reduction of HAZV replication and immune-activation in HuR KO cells can indicate a broad down regulation of many cellular pathways. Or, the inhibition of HAZV replication, due to the HuR KO, could stimulate partially the immunity. The comparison with JEV could help to clarify the results.

→We thank for the suggestion. As mentioned by the reviewer, innate immune suppression due to HuR deficiency was expected to enhance HAZV replication; however, the HAZV titer in HuR KO cells was lower than that in WT cells. As suggested by reviewer, we conducted JEV infection to WT and HuR KO cells and measured cytokine expression (Supplemental Figure 2). Only *Tnf* and *Ccl2* expression after 10 MOI JEV infection at 9 h was slightly increased, suggesting that JEV strongly suppressed the innate immune response. We also stimulated RAW264.7 cells with 1 MOI JEV, however increase of cytokine gene expression was not detected. We have included these points in line 313-317. We understand the concerns that other cellular pathways regulated by HuR may alter HAZV replication. We have included the reviewer's point in the discussion and toned down our conclusion in line 510-512.

Line 262. There is not information about the generation and characterization of the mutated MEF cell lines.

→We have included the information of MEF cells in Materials and methods, lines148-150. *Rig-I/Mda5* KO MEFs, *IPS1* KO MEFs and *Tbk1/Ikk-i* KO MEFs were generated from each KO mice and were kindly provided by Dr Shizuo Akira (Osaka Univ.).

Why did the Authors use different cell lines for the experiments related to figures 3B and 3C? The poly I:C treatment should be included as additional control in figure 3B.

→MEFs are commonly used for analyze innate immune response, and contribution of *Rig-I/Mda5*, *IPS1* and *Tbk1/Ikk-i* during virus infection was confirmed with MEFs (doi: 10.1038/nature04734. doi: 10.1084/jem.20060792. doi: 10.1084/jem.20040520.). Signaling molecules in innate immunity are shared within various cells including MEFs and RAW264.7 cells. We able to utilize these KO MEFs which were established from KO mice. Based on these reasons, we used MEFs to investigate innate immune response after HAZV infection. As reviewer suggested, we included the poly(I:C) treatment in Figure 3B and 3C. MEFs were treated with poly(I:C) for 6 h and medium was replaced. Then, MEFs were infected with 0.1 MOI HAZV, and the virus titer (Figure 2B) and the copy number inside cells (Figure 2C) at 48 h post infection were measured. The virus tier in WT MEFs was reduced by poly(I:C) treatment, but virus titers in *Rig-I/Mda5*, *IPS1* and *Tbk-1/Ikk-i* KO MEFs were not altered by poly(I:C) treatment. These results supported innate immune activation suppresses HAZV replication.

Fig4A: considering the type of mechanism under investigation, more details could be obtained evaluating the amount of positive and negative strand segments of the viral genome using specific primers for retro-transcription and/or the evaluation of viral mRNA production to discriminate between replication and transcription.

→We thank for the suggestion. We performed the experiment according to reviewer's suggestion. WT and HuR KO RAW264.7 cells were infected with HAZV and RNA was isolated at 48 h post infection. HAZV RNA was transcribed using specific primers for positive and negative strands of S, M, and L segments, and the copy number was calculated. The copy numbers of positive and negative strands of each segment in HuR KO cells, except positive strand of M segment, were lower than that in WT cells. We have described this in line 360-365.

Lines 281-284. An additional control should be included, such as the western blot for the N protein of the virus. This could be useful also for data in figure 4D-4E. Viral protein is more stable than RNA and can work as internal control.

→Thanks for the suggestion. We performed western blotting against HAZV N protein to test its stability in Figure 4G. WT and HuR KO cells were infected with 0.1 MOI HAZV, and actinomycin D was treated for indicated time on day2 post infection. We have described this in line 380-382. A comparison of infected cells with non-infected control cells is shown in revised Figure 4E.

Lines 316-318. Authors report the presence of two AU- U-rich sequences in the segment L. What is the situation of the segments S and M? Are there the same or similar sequences? If not, can the potential effect of the deletion of HuR modify the amount of S segment or viral RNA polymerases expression?

→We have added the sequence of CCHFV 3'-NCR in S and M segments in Supplemental Figure 4 and highlighted AU-rich sequences that are not located in the panhandle structure.

Are the mutations performed in both the genome extremities allowing the formation of the panhandle structure?

→Region1 is located 30-40 base pare away from the panhandle structure; therefore, it does not seem to directly affect the formation of the panhandle structure; however, region1 may interfere with formation of the panhandle structure and association with the L protein. We have included this in discussion lines 498-499.

Lines 327-330. Experiments with the inhibitor of HuR should include HAZV replicon and the mutated sequence (delta 1 and delta 2) thus allowing a better comparison.

→To test the inhibitory effect of HuR on HAZV minigenome replication, HEK293 cells were treated with CLMD-2 after transfection, and the luciferase intensities were measured in Figure 5I. CLMD-2 treatment lowered HAZV minigenome replication. We then treated HEK293 cells with CLMD-2 after transfection with Δregion1 and Δregion2 reporter RNA in Figure 5J. Δregion2 lowered luciferase intensity by CLMD-2 treatment, however Δregion1 did not altered.

We thank the reviewer for his/her helpful comments which have allowed us to improve the paper. The following changes have been made.

Reviewer #2:

**Methods**
1. The study objectives are clearly stated
2. The exact experimental details (cell line, MOI, time point post infection/transfection/stimulation) are sometimes somewhat difficult to find. Recommend putting all these details in both the Figure legends and the Methods section, and in the main text where appropriate.

→We apologize unclear discretion of the methods. Based on the reviewer’s comments in the following, we have edited the manuscript and added experimental details in the Figure legends.

3. In Figure 4 authors assess viral RNA stability using Actinomycin D. Actinomycin D blocks cellular transcription, but I am not certain if it affects replication of negative strand RNA viruses like HAZV. It certainly does not affect replication of +RNA virsues as ActD is routinely used to label viral RNA in the absence of host transcription. Since ActD is well tolerated by most cell lines, authors should add some later time points and viral titers to more convincingly show that ActD inhibits viral replication. The presented data using ActD shows some inconsistencies, as in Fig 4D the HAZV RNA levels remain steady during 4h ActD treatment, whereas in Fig 4E HAZV RNA levels decreased in ActD treated infected WT cells.
→We treated the cells with 2.5 μg/mL actinomycin D for a longer period; 8h, and replaced Fig.4H and 4I. Our data support that RNA of all three segments is destabilized in HuR KO cells compared to that in WT cells (Fig.4I). Actynomycin D is an anticancer drug that kills target cells by binding to guanidine residues. We investigated cell viability using the LDH assay and N protein expression during actinomycin D treatment to support Figure 4H and I. LDH concentration in the supernatant did not increased for 8h, after 2.5 μg/mL actinomycin D treatment, suggesting that the cells were viable under these conditions. RAW264.7 cells at 48 h post-infection with HAZV were treated with actinomycin D for the indicated time, and N protein expression in WT and HuR KO cells was constant during the treatment (Fig.4G).
We then tested whether HAZV replication was inhibited by actinomycin D. RAW264.7 cells were treated with the indicated concentration of actinomycin D for 48 h, and the release of LDH was measured (Supplemental Fig.3B). Treatment with 200 ng/mL actinomycin D induced LDH release, whereas treatment with < 20 ng/mL actinomycin D did not increase LDH concentration. RAW264.7 cells infected with HAZV were treated with the indicated concentrations (< 20 ng/mL) of actinomycin D, and the virus titer was measured 48 h after infection (Supplemental Fig.3C). We found that HAZV titer was lowered by actinomycin D treatment. We have included these points in lines 389-396.

**Results**

Reviewer #2: 1. While I don’t doubt that HuR plays a role in the HAZV replicative cycle, the data demonstrating that HuR is supporting HAZV/CCHFV replication by directly binding and stabilizing viral RNA is not very strong, especially the mechanistical side of it. HuR KO only has a modest effect on viral titers (0.5-1.0 log reduction), and knock-in experiments demonstrating that ectopic expression of HuR relieves the negative effect of its depletion have not been done. A lot of the presented data is somewhat circumstantial. E.g. authors state that HuR bind directly to HAZV RNA, but since these are IP experiments using cell lysates, it could be a protein complex binding to the RNA.

→We thank for the suggestion. As the reviewer pointed out, we did not perfom the knock-in experiments in our previous manuscript. We have included the results of minigenome reporter assay with HuR expression (Figure 4D and 5B). HAZV (Figure 4D) and CCHFV (Figure 5B) minigenome reporter activity was increased by HuR expression. We agree with the reviewer's point that we did not directly show HuR and CCHFV genome RNA interaction. To tone down HuR and RNA interaction, we have changed “bind” to “association” or “interaction” in line 61, 130, 134, 400,473,483, 488,498, 513 and 516, and we have deleted “bind” in tile of Figure 4 (line 782). We have included the sentence to tone down the interaction between HAZV RNA and HuR; “We have shown by IP experiments using cell lysates that other proteins may be involved in RNA-HuR complex formation.” in lines 506-507.

2. Including the section in the introduction addressing innate immunity (line 81-90) and Fig. 2+3 does not fit well with the HuR/HAZV story, resulting in a disjointed manuscript. The premise presented in Fig. 2, describing that depletion of HuR results in dampened immune responses was already published by the authors, so this is not novel and not specifically related to HAZV infection. Additionally, this does not explain the mechanism(s) by with HuR supports HAZV replication, as the expectation is that lower immune responses would result in higher HAZV titers.

→As reviewer mentioned that “The expectation is that lower immune responses would result in higher HAZV titers”, it was unclear why innate immune activation by HAZV infection was not involved in its replication. As shown in Figure 3A, we found for the first time that innate immune activation by HAZV infection is dependent on the RIG-I/MDA-5 signal transduction pathway. Innate immune response in WT MEFs after HAZV infection was increased, but not in *Rig-I/Mda5*, *IPS1,* and *Tbk1/Ikk-i* KO MEFs (Figure 3A). And HAZV replication in WT MEFs was similar to that in these KO MEFs, indicating that innate immune activation by HAZV infection did not alter viral replication (Figure 3B, C). On the other hand, pre-poly(I:C) stimulation lowered viral replication in WT MEFs, but not in those KO MEFs (Figure 3B, C). Pre-poly(I:C) stimulation is known to induce robust activation of innate immune response, whereas innate immune activation by HAZV is weakened by viral proteins. It has been reported that OTU domains of CCHFV and HAZV interfere with host innate immune system via the ubiquitin protease activity (doi:10.1016/j.celrep.2017.08.040, 10.1107/S2059798320012875 ) and HAZV N protein interferes with the binding of TRIM25 to RIG-I and subsequent activation of RIG-I (10.3390/v14091965). In addition, pre-poly(I:C) stimulation suppressed HAZV replication in both WT and HuR KO cells (Figure 3D,E). These results suggest that robust innate immune activation by pre-poly(I:C) stimulation lowers HAZV replication; however, weak innate immune activation by HAZV infection does not affect its replication.

We have clarified these points in the result line 332-353 and the discussion line 453-469.

3. Fig 3: while interesting in itself, this figure does not contribute to the HuR story and does not fit in well with the data presented in Fig 1, 4 and 5.

→This is the same answer as commented in the upper section.

4. Demonstrating the interaction of HuR with HAZV/CCHFV RNA is a pivotal figure in this manuscript (Fig 4C, Fig 5B/E). Authors pull down viral RNA from WT and HuR KO cells using a HuR antibody. The presented difference looks impressive but obviously the absence of HuR in the KO cells will preclude any RNA immunoprecipitation. Additionally, authors already demonstrated that viral RNA levels are lower in HuR KO cells. Authors should include antibodies against NP as a positive control to demonstrate that HAZV/CCHFV RNA is indeed present in somewhat similar levels before IP.

→We thank for the suggestion. We performed WB against NP after 48 h HAZV infection and NP expression in WT and KO RAW264.7 cells was similar level (Figure 4E).

5. Does CLMD-2 inhibit HAZV infection?

→RAW264.7 cells were treated with CMLD-2 after infection with HAZV and virus titer after 48 h infection was measured (supplemental Figure 5). HAZV titer was lowered by CLMD-2 treatment.

**Conclusion**

Reviewer #2: 1. Since the majority of the experiments are done with Hazara virus, the title/abstract/author summary/introduction should reflect this. Only the last figure uses CCHFV, and this is restricted to the use of a minigenome assay (i.e. no infectious CCHFV was tested).

→We agree with the reviewer's point. We have changed the title, abstract, author summary and introduction. We have changed title to “HuR (ELAVL1) regulates the CCHFV minigenome and HAZV replication by associating with viral genomic RNA”, which included reviewer’s point. We have also included limitation of this study to mention that we did not used CCHFV infection model at the discussion in line 522-524.

2. The discussion section is largely repeating the results section, rather than placing their results in a larger context. HuR has been implicated in the replicative cycles for many viruses (including JEV). This should be more extensively addressed in the Discussion.

→We have changed in the discussion in line 438-452 to mention about JEV and TBEV.

3. Authors do not address limitations of their study in the Discussion.
→We have included the limitation of the study in lines 520-524.

**Editorial and Data Presentation Modifications?**

Reviewer #2: 1. Overall, this manuscript would benefit from substantial rewriting. The English language is in places insufficient. Various sentences are unclear or confusing.

→We apologize for English writing and we applicate these comments and suggestions. We have edited the sentences based on the point by point comments as follows.

2. Please order the methods section in order of appearance in the manuscript

→We have edited order of the methods in “Materials and methods”. Order is following, Cells and reagents, Generation of mutant cell line, Virus and viral titration, RNA isolation, RT-PCR assay, ELISA, Western blotting, Promoter reporter assay, Minigenome assay, RNA stability assay, RNA immunoprecipitation, Statistical analysis.

3. Line 133: Please provide more information on the TBEV/JEV strains used

→Line 174-176, we have added the strain information of JEV and TBEV with references.

4. Line 139-159: please separate this into separate sections describing RNA isolation, the RNA stability assay and RT-PCR assay.

→Line 183-198 and 246-248: We have separated section to RNA isolation, RNA stability assay and RT-PCR assay.

5. Line 146-159: please provide the primer sequences as a supplementary table

→Line 187-198: we have deleted the primer sequences from “RT-PCR assay”. Primer information is provided in S2 table.

6. Line 162 (ELISA methods section): please specify here what cells were used.

→Line 201: we have change “Cells” to “RAW264.7 cells”.

7. Line 180: please indicate used MOI and how long after infection luciferase activity was measured.

→Line 323-324: we have added MOI and time of measurement. We performed luciferase assay at 24 h after 1 MOI HAZV infection.

8. Line 224-226: The results section starts very abrupt. It would be helpful if it started with a statement of what/why this was done. It is unclear why MEF cells were infected alongside RAWs.

→We thank for the suggestion. Line 266-271: We have included the sentence that explain the reason for macrophages and MEFs.

9. Figure 1: The absence of HuR expression was not confirmed for the RAW264.7 cells.

→We included a result of WB by anti-HuR antibody in Figure 1C to confirm HuR deficiency and mentioned about it in line 284-285.

10. Line 239-241: please rewrite to enhance clarity

→Thank you for pointing out. We have deleted the sentences in the revised manuscript.

11. Line 242-245: It is unclear how HAZV/CCHFV OTU domains are linked to HuR and why authors looked at the induction of various immune genes.

→We thank for the suggestion. Line 298-301, we have edited the sentence to mention about innate immune response and line 242-245 in old manuscript was omitted. We have discussed about HAZV/CCHFV OTU domains in the discussion.

12. Line 246: please specify that RNA levels are measured

→Line 303-305: we edited the sentence as “expression of *Il6*, *Ifnb*, *Tnf*, *Cxcl10*, *Il10*, *Il12p40*, *Ccl2* and *Tgfb* relative to non-infected control cells and the copy number of S segment inside cells were measured”.

13. Fig 2A should also assess HAZV RNA levels.

→We have measured S segment RNA levels after infection in revised Figure 2B.

14. Fig 2A: It is unclear what the y-axis reflects. Is this fold increase compared to uninfected cells?

Same question for Fig 2E.

→Yes, Y-axis in Fig.2A shows a fold increase compared to non-infected control WT cells. Line 758-760: we added the explanation in the Fig.2 legend. We have changed label “control” to “non-infected control” and changed label of y-axis “Relative to non-infected control” in Fig.2A.

→Y-axis in Fig.2F,G (same as Fig.2E in the previous manuscript) is a ratio of the intensity of firefly luciferase (IFN-β/ISRE promoter) to the intensity of renilla luciferase (internal control promoter). Line 767-769: we added the explanation in the Fig.2 legend.

15. Fig 2C: HuR protein levels seem to be upregulated in infected cells? This would be interesting to pursue more.

→It was reported that HuR expression is increased by the stimulation with poly(I:C) (doi:10.1002/eji.201444979). HAZV also activated innate immunity through RIG-I/MDA5 dependent manner as same as poly(I:C). Increase of HuR expression may support HAZV replication. We discussed about HuR expression in lines 510-512.

16. Fig 3A: it is unclear how long after HAZV infection gene expression levels were determined, or what MOI was used.

→MEF cells were infected with 1 MOI HAZV and gene expression was measured at 24 h after infection. We have included the information in the manuscript (line 323-324) and the Fig.3 legend (lines:773-775).

17. Fig 4: Relative viral RNA levels are relative to what? Control is uninfected?

→We have changed calculation of relative viral RNA levels in the revised manuscript. We calculated copy number based on referenced DNA. Y axis was changed to “Copy number / 1 μg RNA” in Fig.1E, Fig.2B, Fig.3C,E, Fig.4A,B,F, Fig5.C,D,F and was changed to “Copy number / precipitant” in Fig.4F.

**Summary and General Comments**

Reviewer #2: Authors investigated the role of RNA-binding proteins during Hazara virus infection by generating 66 KO cell lines using CRISPR. HuR was identified as one of the strongest hits, of which depletion resulting in a modest reduction in HAZV titers. As the authors described in a previous publication, depletion of HuR results in an impaired activation of immune responses. Furthermore, they show that both HAZV and CCHFV minigenome activity is affected in HuR KO cells, likely due to the lack of HuR-mediated viral RNA stabilization.

We thank the reviewer for his/her helpful comments which have allowed us to improve the paper. The following changes have been made.

Reviewer #3:

**Methods**
Reviewer #3:

line 206-217: refer somewhere to S1 table

→line 165, we inserted the sentence that referred table S1 in the material and methods.

Fig 3c: Add in the method the qty of poly(I-C) used, how long was the pre and post-treatment, when did you recover the supernatant? how did you purify the supernatant from poly(I-C) before titration to avoid the poly(I:C) to activate the cells you were using for your titration?

→We have added the protocol for poly(I:C) stimulation in lines 145-147. We thank the reviewer for this suggestion. In the previous manuscript, we did not consider poly(I:C) contamination during the measurement of the virus titer. We have replaced Figure 3B,C,D,E. RAW264.7 cells were stimulated with poly(I:C) for 6 h and infected with 0.1 MOI HAZV after replacing the medium with fresh medium. We omitted post poly(I:C) stimulation, because it is difficult to separate poly(I:C) from HAZV in the supernatant.

several figures: Explain in the method how you calculated the relative RNA binding: do you have a standard RNA for your RT-PCR to determine the number of copies? Or?

→We have calculated copy number based on standard RNA/DNA and replaced or added Fig.1E, Fig.2B, Fig.3C,E, Fig.4A,B,F, Fig5.C,D,F. We have included the method in lines 187-198.

Please note the n for each figure in the legend. By eyes, it looks like that the number of repeat in the same experiment is not the same sometimes. For example, Fig 5B, anti HuR, it looks like n=6 for the positive strand while it seems to be 4 in the negative strand. Fig 5G, it seems that n=4 for control but n=3 for the treated ones. If it is the case, please repeat the experiments to have the same number of repeat for each condition.
→As reviewer pointed out, number of sample in each group was differ in Fig.5B and G in the previous manuscript. We have performed the same experiment with the same number in each group and replaced Fig.5C (n = 6, same as Fig.5B in the previous) and 5G (n = 6) in the revised manuscript.

**Results**
Reviewer #3:

Figure 1: what about HAZV RNA level inside the cells?

→RNA was isolated from WT, KO1 and KO2 cells that infected with HAZV at day 2, and copy number / 1 μg RNA was calculated (Fig.1E). The copy number in HuR KO cells was lower than that in WT cells.

line 224: please explain why you made a kinetic of hazv infection on raw264.7 and MEF

→Monocytes and macrophages are infected with CCHFV, which may contribute to disease progression and excessive inflammatory response. We are interested in relation between CCHFV (or HAZV) and immune response, therefore RAW264.7 cells was used for model of macrophage. MEF used as a representative of non-immune cells. We included these points in line 266-271

line 227: viral titer doesn’t decrease at 72h for raw264.7

→As reviewer mentioned, viral titer in RAW264.7 cells at 72 h was almost equal to viral titer at 48 h. We have edited as “The virus titers of RAW264.7 cells peaked at 24-48 h and sustained to 72 h (Fig.1A). The virus titers of MEFs peaked at 48 h and decreased at 72 h (Fig.1A).” in line 271-273.

line 229: confusing. move and rephrase the part “and virus titer...plaque assay” after “...S1 table.” and move “fig 1B” after “highlighted” line 233

→We thank for the correction. We moved “and virus titer...plaque assay” to line 276-277 and “Fig 1B” to line 279, as the reviewer indicated.

line 232: is it “both” cell lines or “the” cell lines? it seems it is “the” cell lines.

→Thank you for pointing out. We have change “both” to “the” in line 278. The sentence was edited as “The virus titers of these cells were plotted, and the cell lines that showed higher or lower titer than the WT RAW264.7 cells were highlighted. (Fig.1B).”

line 234: why did you choose only these hits?? it is not because they were the highest or the lowest compare to wild-type as for example, they are 2 cells lines between.

→We have shown that HuR regulates the innate immune response by binding to the 3’-NCR of host target mRNA, and we are interested in the regulatory role of HuR. To investigate whether HuR regulates the stability of exogenous RNA in addition to the host mRNA, we selected HuR for this study. As the reviewer indicated, other hits potentially regulate HAZV and CCHFV RNA. In the future, we will investigate the role of molecular mechanisms in other RNA-binding proteins. We have included this in lines 280-284.

line 235: please precise if JEV and TBEV are control and explain why you choose them as control

→It was reported that HuR exhibits an antiviral effect against ZIKA virus (doi: 10.1128/JVI.00520-19). JEV and TBEV are flavivirus, which are the same genus with ZIKA virus. Therefore, JEV and TBEV use as control for HAZV. We have discussed in line 438-440 in the discussion.

line 236: add “48hpi” after “were measured”

→We applicate the indication. Based on the reviewer 1 comment, we have combined Fig. 1C to Fig.1D and have deleted line 236 in the previous manuscript.

Line 238: you used JEV and TBEV and show the data but nothing is discussed. Please discuss the data, or you remove the data.

→We thank for the suggestion. We have included discussion in line 438-452.

Line 242: what about the other hits? why did you choose only HuR? Explain

→We have shown that HuR regulates the innate immune response by binding to the 3’-NCR of host target mRNA, and we are interested in the regulatory role of HuR. To investigate whether HuR regulates the stability of exogenous RNA in addition to the host mRNA, we selected HuR for this study. As the reviewer indicated, other hits potentially regulate HAZV and CCHFV RNA stability. In the future, we will investigate the role of molecular mechanisms in other RNA binding proteins. We have included this in lines 280-284.

Line 245-248/fig 2A: Why did you infect with MOI 1 for 9h while until now you used MOI 0.1 and 48hpi? Even in your kinetic on wt and ko cells you do not have 9h time-point.

→To investigate viral replication in Figure 1, the viral titer was measured after 0.1 MOI HAZV infection. Whereas, cells were infected with 1 MOI HAZV to investigate innate immune response in Figure 2 and 3. If cells were stimulated with 0.1 MOI, only 1/10 cells were stimulated by the initial infection, and it was difficult to detect cytokine response at 9h. In addition, we must wait until a sufficient number of cells are infected after viral replication to detect innate immune response. However, if the viral replication cycle is lowered by the deficiency, the amount of replicated virus is lowered, which is expected to decrease the innate immune response. It is difficult to determine whether the innate immune response or viral replication is regulated by the target gene. Therefore, cells need to be stimulated simultaneously by 1 MOI to investigate the innate immune response.

As the reviewer pointed out, we measured gene expression at 9h time-point. HAZV infection induced the production of cytokines, such as IFNβ and IL-6, as shown in Figure 1A. These produced cytokines may reactivate cells at 48 h post infection, which is known as the autocrine effect. To avoid the autocrine effect, we measured cytokine levels at an earlier time point 9 h post infection in RAW264.7 cells. *Il6* gene expression was detectable after HAZV infection at 9 h post infection; however, IL-6 production in the supernatant at 9 h post infection was not detectable revised Figure 2C.

Line 248-250: you changed again the MOI to be back to MOI 0.1 and here you go for day 1, 2 and 3pi. Nothing can be compared as MOI 1 9h, there is a decrease in IL-6 in KO cells while at MOI 0.1 the significant decrease is 3dpi. Both data together seems not to match.
Line 250-253: Back to MOI 1.

→We measured IL-6 production at 9 h and 24 h after 1 MOI HAZV infection and have replaced Figure 2C. Although *Il6* expression was increased at 9 h by RT-PCR (Figure 2A), IL-6 production at 9 h was undetectable. IL-6 production increased at 24 h and lowered in HuR KO cells.

line 253: IRF3 phosphorylation level begun to increase from 9h in wild-type cells but was less increased in HuR KO cells. Then you show data for IRF3, pp65 and p65 but nothing in the text. please write a sentence to give results for them.

→We thank for pointing out. We have mentioned phosphorylation of p65 in line 320.

line 255: Why changing the cells to hek293? please explain. Then there is no data on the kinetic of the virus on these cells. Which MOI and why 24pi?

→RAW264.7 cells were technically difficult to efficiently transfect plasmid. Only 1-3 % of RAW264.7 cells express target gene after transfection, however more than 70 % of HEK293 cells express target gene after transfection. To perform *Ifnb* promoter assay and mini-genome assay in Figure 4 and 5, we have generated HuR KO HEK293 cells. We apologize not clearly mentioned the method of promoter assay. Cells were infected with 1 MOI HAZV and measured at 24 h after infection. IFN-β and ISRE promoter assay commonly performed next day after poly (I:C) transfection. As we explained in upper comment related line 227-233, an earlier time point is better rather than 48 h after infection. Therefore, we performed next day after HAZV infection and poly (I:C) stimulation in this protocol.

line 257: not suppressed but reduced.

→We have changed “suppressed” to “reduced” in line 323-324.

There is a lack of logic in your data: you show data for several cytokines, then you focus on Il-6 (why particularly Il-6?) then you go to IRF3/p65 to come back to IFN-β. What about TNF, Cxcl10 or Ccl2??

→Inflammation causes disease progression during CCHFV infection. IL-6 is one of an important cytokine that controls inflammation. As the reviewer mentioned, other cytokines such as IFN-β, TNF, Cxcl10 and Ccl2 also expected to show reduced production in HuR KO cells. We chose IL-6 as a representative cytokine gene in RAW264.7 cells to monitor innate immune activation. HEK293 cells are commonly used for promoter assays because of their high transfection efficiency; however, it is known that HEK293 cells do not produce IL-6 after innate immune activation. IFN-β promoter contains an IRF-binding site, and Figure 2C shows that IRF3 phosphorylation was reduced in HuR KO cells. We have performed IFN-β promoter assay to support innate immune activation by HAZV infection. To address the reviewer’s concern, we performed *Il6* promoter assay in WT and HuR KO HEK293 cells; however, *Il6* promoter activity was not clearly increased after HAZV infection (attached Figure). *Il6* promoter may not function in HEK293 cells for unknown reasons after HAZV infection and poly(I:C) stimulation. Instead, we performed Interferon Stimulated Response Element (ISRE) promoter assay, which contained IRF binding site in Figure 2G, and we observed similar result with *Ifnb* promoter assay in Figure 2F.

line 259: HAZV infection triggers activation of innate immune response in mouse macrophages.

→We thank for the correction. We have edited the sentence in line 327-328 as indicated.

line 260: “...to be recognized by RIG-I/MDA5”. Add “and HuR regulates innate immune response via RIG-I/MDA5-dependent... pathways” that is already noted line 242 to make it more understandable.

→We have edited 327-329 as “HAZV infection triggers activation of innate immune response in mouse macrophages (Fig.2) and genomic RNA in negative-stranded RNA viruses has been reported to be recognized by RIG-I/MDA5.”.

line 261/fig 3A: why do you change again the cell line? note on the figure that – and + mean – infection or + infection. Which MOI? note in the legend the MOI and the time-post-infection. Why 24h while the pick of viral titer in MEK is 48hpi? Explain.

→Innate immune signaling molecules are conserved in various type of cells and MEFs are widely used to analyze innate immune response. We are available KO MEF line derived from KO mice. We have included the information of MEF cells in Materials and methods, lines 148-151. *Rig-I/Mda5* KO MEFs, *IPS1* KO MEFs and *Tbk1/Ikk-i* KO MEFs were generated from each KO mice and were kindly provided by Dr Shizuo Akira (Osaka Univ.). We apologies not clearly mention the method in Fig. 3A. MEFs were infected with 1 MOI HAZV and gene expression was measured at 24 h post infection. We are included method in line 335-336 and Fig. 3 legend.

MEFs incudes production of cytokine by HAZV infection as shown in Fig. 3A. Produced cytokines such as IFN-β and IL-6 may re-activate cells (autocrine effect). Innate immune signaling molecules and cytokine signaling molecules overlapped each other. To avoid autocrine effect, it is important to measure cytokine expression at earlier time point. We have measured cytokine at 9 h post infection in MEFs, however increase of gene expression was not detectable. MEFs are non-immune cells, and it is suspected that cytokine expression is lower and the response against viral RNA is slower than in immune cells. From these reasons, we measured cytokine gene expression at 24 h post infection after 1 MOI HAZV infection.

line 264: significantly increased in WT infected cells contrary to KO cells

→We have edited the sentence in line 336-337 as indicated.

Figure 3B: back to MOI 0.1 48hpi. what about the RNA inside the cells?

→We measured HAZV RNA levels inside cells (Fig.3C) and obtained results similar to those in Fig.3B.

line 268: indicating the innate immune response doesn’t affect virus production.

→We have edited the sentence in line 343-344 as indicated.

line 269/fig 3c: back to raw264.7. why? Also explain the role of poly-(I:C). Add in the method the qty of poly(I-C) used, how long was the pre and post-treatment, when did you recover the supernatant? how did you purify the supernatant from poly(I-C) before titration to avoid the poly(I:C) to activate the cells you were using for your titration? Please note statistical analysis comparing pre and post poly(I-C) treatment. It seems the data for HuR KO cells are not significant, you cannot say that “pre-stimulation with poly(I-C) in both WT and HuR KO cells reduced the virus titers compared to the control or post-poly(I-C) (line 272). Please rectify.

→We apologize for unclear description of the experiment and method related to Fig.3C in the previous manuscript. We found that poly(I:C) stimulation in MEF cells suppressed viral replication in revised Fig.3B,C. We tested whether poly(I:C) stimulation suppresses HAZV replication in RAW264.7 cells WT and HuR KO cells, similar to MEFs (Fig.3D,E). RAW264.7 cells were stimulated with poly(I:C) for 6 h, and were infected with 0.1 MOI HAZV after replacing fresh medium. The virus titer in the supernatant was measured at 48 h after infection. We did not consider poly(I:C) contamination during the measurement of the virus titer in the previous manuscript. We have replaced Fig.3C in the previous manuscript to Fig.3D,E in the revised manuscript. As mentioned by the reviewer, pre-poly(I:C) stimulation is not significantly lower than post-poly(I:C) stimulation and it is difficult to separate poly(I:C) from HAZV in the supernatant. We omitted post-poly(I:C) stimulation in revised Fig.3D,E. We have added the protocol for poly(I:C) stimulation in lines 145-147.

Lowered innate immune activation by HuR deficiency after HAZV infection (Fig.2) expects increase of HAZV replication. It was unclear why innate immune activation by HAZV infection was not involved in its replication. As shown in Fig.3A, we found that innate immune activation by HAZV infection is dependent on the RIG-I/MDA-5 signal transduction pathway. Innate immune response in WT MEF after HAZV infection was increased, but not in *Rig-I/Mda5*, *IPS1* and *Tbk1/Ikk-i* KO MEF (Fig. 3A). In contrast, HAZV replication in WT MEF was similar to that in those KO MEFs, indicating that innate immune activation by HAZV infection did not alter viral replication (Fig.3B,C). On the other hand, pre-poly(I:C) stimulation lowered viral replication in WT MEF, but not in those KO MEF (Figure 3B,C). Pre-poly(I:C) stimulation is known to induce robust activation of innate immune response, whereas innate immune activation by HAZV is weakened by viral proteins. It has been reported that OTU domains of CCHFV and HAZV interfere with host innate immune system via the ubiquitin protease activity (doi:10.1016/j.celrep.2017.08.040, 10.1107/S2059798320012875) and HAZV N protein interferes with the binding of TRIM25 to RIG-I and subsequent activation of RIG-I (doi:10.3390/v14091965). In addition, pre-poly(I:C) stimulation suppressed HAZV replication in both WT and HuR KO cells (Fig.3D,E). These results suggest that robust innate immune activation by pre-poly(I:C) stimulation lowers HAZV replication; however, weak innate immune activation by HAZV infection does not affect its replication. We have described these points in the result line 327-353 and in the discussion line 453-469.

line 270: is there any paper showing that the protocol you used for innate immune response stimulation really stimulate the immune response. if yes, please cite. if not, please measure the cytokines response under poly(I-C) treatment.

→Yes, we have measured cytokine gene expression in HuR KO cells after poly(I:C) stimulation. We cited 31 (doi: 10.4049/jimmunol.1701282) in line 349.

line 280/fig 4B: you changed again the cells to Hek293. Please justify. Change “to test” by “to confirm”.

→Only 1-3 % of RAW264.7 cells express target gene after transfection, however more than 70 % of HEK293 cells express target gene after transfection. To perform minigenome assay, transfection efficiency is important to detect luciferase activity, therefore we have generated HuR KO HEK293 cells in Figure 2E and performed minigenome assay in Figure 4C in the revised manuscript. We have changed “to test” to “to confirm” in line 366.

line 289: “was not precipitated” to change by “slightly” or similar. Explain in the method how you calculated the relative RNA binding: do you have a standard RNA for your RT-PCR to determine the number of copies? Or?

→We changed “was not precipitated” to “was slightly precipitated” in line 378. We have calculated copy number per precipitant based on standard DNA of S, M, and L segments and replaced Figure 4F in the revised manuscript. We have included the method in lines 194-198.

Line 295: control cells showed an increase in viral RNA amount whereas ... suppressed viral RNA replication” or “de novo synthesis”.

→We have changed the sentence to “Untreated control cells showed an increase in viral RNA, whereas 2.5 μg/mL actinomycin D-treated cells showed a gradual decrease in viral RNA” in line 386-389.

Line 316: “NCR in L segment...”. But you used the S segment for HAZV previously. If it is for all NCR please change “L segment” to “L, S and M segments” or similar.

→We have changed “L segment” to “L, S, and M segments” in line 417 and added Supplemental Fig.4 showed sequence of 3’-NCR in CCHFV S and M.

Line 327: on CCHFV mini-replicon replication and not on CCHFV replication. Same line 330

→We have inserted “mini-replicon” in line 427-428 and 430.

**Conclusions**
Reviewer #3:

The discussion has to be modified: in general, the discussion needs to go a bit deeplier and try to give more explanation then just repeating the results. More of that, the conclusions go to far and have to be tempered.

→We have edited the discussion as indicated in the red letters in the revised manuscript. We have included discussion of JEV and TBEV in line 438-452, and discussion of viral replication after innate immune activation in line 453-469. We have tone down the discussion by changing “binding” to “association” in 473,483, 488, 498-499, 513, and 516, and adding the limitation of study in line 510-512 and 520-524.

Line 333: we found that HuR binds to the AU-rich region of the 3’-NCR of the L segment of CCHFV in a mini-replicon system.

→line 434-435, We have changed that sentence as “we found that HuR binds to the AU-rich region of the 3’-NCR of the L segment of CCHFV in a mini-replicon system.”.

line 337: not suppress but reduce

→We thank for pointing out. We have rewritten discussion line 438-452 based on the previous comment and we have deleted related sentences.

line 338 “it increasesd JEV replication” And? any explanation?

→line 438-452, we have included discussion related JEV replication.

line 342: not suppressed, but “...induce expression of some cytokine gene, which were strongly reduced by HuR deficiency”

line 345: “comparable to wt cells” instead of “comparably increased.”

line 348-350: it is results but no explanation. please give some possible explanation.

→Thank you for pointing this out. We have revised the discussion based on the comments in the upper section. We have omitted these sentences from the revised manuscript.

line 381: significantly reduced, not suppressed.

→We have changed “suppressed” to “reduced” in line 500.

line 388: it is not required, it participates.

→We have changed “required” to “participates” in line 515.

line 390: CLMD-2 doesn’t suppress CCHFV minigenome replication but reduces (50% decrease is far from suppression)

→We have changed “suppressed” to “reduced” in line 517.

line 391: could be new therapeutic target.

→We have changed “may be a potential therapeutic strategy” to “could be new therapeutic target” in line 519-520.

line 392: finish the discussion by explaining that all the findings have to be validated in a real infection study using CCHFV.
→We have added “these findings need to be confirmed with CCHFV infection” in line 523-524.
--------------------

**Editorial and Data Presentation Modifications?**

Reviewer #3: Abstract Rephrase abstract lines 33-36 as it is not clear from cchfv to hazv even if you say that hazv used as a model before and is that the screening that was done using a mutant cells lines? please make it clearer (the author summary is clearer)

→Thank you for the suggestion. We have changed Abstract that included the strategy of screening.

Author summary
Line 56: In the author summary: hur inhibitor doesn’t suppress cchfv minigenome replication. 50% is not suppressing, it is reducing

→We have changed “suppressed” to “reduced” in line 63.

Introduction

Line 111: HuR is not required for the replication of CCHFV: you still have 25 to 50% luciferase activity in KO cells (fig 5A)

→W have changed “required” to “regulates” in line 131.

**Summary and General Comments**
Reviewer #3: This manuscript is of interest for researchers in CCHFV and give interesting preliminary data for a possible way to explore in antiviral development. Nethertheless, some modifications are required.
The titer has to be modified as you do not show that HuR supports (real) CCHFV viral replication, and please precise that is in a mini-replicon system. You can also add HAZV in the title as it is 80% of your data...

→As the reviewer pointed out, HAZV used as model virus of CCHFV and CCHFV replication only confirmed with a minigenome system. We have changed the title to “HuR (ELAVL1) regulates the CCHFV minigenome and HAZV replication by associating with viral genomic RNA” to be tempered. We would confirm the contribution of HuR by CCHFV infection model in BSL-4 facility in the future.

Please put p value on top of each batch of sample and not on different batches all together. For example Fig 2A, put the significance for KO1 and KO2 separately.

→We put *p* value in Fig. 2A,B,F, Fig. 5A and Fig. 4C that included KO1 and KO2 in the data.

Why using Raw264.7 and not human cell lines like THP-1? It would have given more relevant data, particularly for the immune response part.

→THP-1 is human monocyte cells and can differentiate to macrophage in the presence of TPA. THP-1 is suitable for analysis of human immune response as the reviewer indicated. We have established strategy for generation of mutant cell using RAW264.7 cells (<https://doi.org/10.1093/intimm/dxae024>), and we applied the strategy in the screening experiment. In this experiment, we confirmed HuR function in mouse macrophage model and we agree that it is important to investigate HuR function in human immune responses. We will test HuR function in human immune cells, including THP-1 in the future.

For all data involving the virus, data on viral RNA level inside the cells are necessary, not only the production of viral particles, to confirm the effect of the KO of HuR in HAZV RNA replication.
→We thank for the suggestion. We have investigated copy number of viral RNA inside cells and added in Fig.1E, Fig.2B, Fig.3C and Fig3E.
