# Supplementary material for: HuR (ELAVL1) regulates the CCHFV minigenome and HAZV replication by associating with viral genomic RNA
Source: PLoS Negl Trop Dis. 2024 Sep 30;18(9):e0012553. doi: 10.1371/journal.pntd.0012553 (PMC11466401; doi:10.1371/journal.pntd.0012553)
Supplement: Attachment — Submitted filename: Response to reviewers_240915.docx [file pntd.0012553.s010.docx]

We thank the reviewer for his/her helpful comments which have allowed us to improve the paper. The following changes have been made.

Comment by Reviewer #1:

Reviewer #1: The methods section of the revised manuscript has improved since the first submission. It is acceptable.

Reviewer #1: Results are clearly presented and match the experimental plan. All the new experiments help to support Authors' hypothesis.

Reviewer #1: Conclusions are supported by results and clearly presented. The content of the conclusions meets editorial requirements.

Reviewer #1: (No Response)

Reviewer #1: The new version of he manuscript is significantly improved and data can support the Authors' conclusions.

I just suggest to check the text for few type errors and some modifications:

line 36: I do not understand why Authors wrote (or the stability of mRNA). This is the same concept reported in the sentence

→We thank for the indication. We have deleted “(or the stability of mRNA)” in line 36.

Fig. 4b: change "psitive" with "positive".

→We have corrected this error in Fig.4b.

lines 366-367: I am not sure that the sentence “The reporter RNA consisting of the luciferase gene with the S segment of the NCR of the HAZV JC280 strain was transfected...” is correct. Probably it is clearer as “The reporter RNA consisting of the luciferase flanked by the NCR of the S segment of the HAZV JC280 strain was transfected...” There are other few sentences with the same form to check.

→We have corrected the sentences as the reviewer indicated in line 366 and 401.

line 452: change "dose" with "does"

→Thank you for pointing out. We have corrected the error.

We thank Dr. Vanessa M. Monteil for helpful comments which have allowed us to improve the paper.

Comment by Reviewer #3:

Reviewer #3: The method part has been completed to give more details.

Reviewer #3: The results part has been deeply corrected/completed to make the data stronger.

Reviewer #3: The conclusions are now better supported by the data and are now reflecting the real scope of the data.

Reviewer #3: (No Response)

Reviewer #3: I would like to acknowledge the authors for taking into account all my comments and having completed/modified the figures/text accordingly. The data are now completed and the conclusions are relevant. The new version of the MS is, in my opinion, ready for publication.
